# The influence of androgynous streamers on consumers’ product preferences

**DOI:** 10.3389/fpsyg.2022.1029503

**Published:** 2022-12-01

**Authors:** Feng Wenting, Xue Shuyun, Yang Ying, Huang Hai

**Affiliations:** ^1^Gemmological Institute, China University of Geosciences, Wuhan, China; ^2^Research Center for Psychological and Health Sciences, China University of Geosciences, Wuhan, China

**Keywords:** the streamers, gender role, attachment theory, gender stereotype, consumers’ preferences, jewelry product

## Abstract

Since the rapid development of network technology, the rise of live-streaming shopping platforms has followed. Some streamers influence consumers’ preferences for products through their gender role attributes, thus generating great commercial value. Based on attachment theory and using an experimental approach, this study explored the impact of streamers’ gender roles (single gender/androgyny) on consumers’ preferences through 2 studies. Study 1 shows the androgynous streamer elicits a higher product preference than the single-gender (masculine and feminine) streamer. Study 2 demonstrates the moderating effect of gender stereotypes through 2 experiments to construct clear boundary conditions for the main effect and the results show that regardless of whether the streamer is male or female when individuals have a high gender stereotype, the single-gender streamer leads to a higher product preference than the androgynous streamer. When individuals have a low gender stereotype, the androgynous streamer promotes a higher product preference than the single-gender streamer.

## Introduction

With the rapid development of network technology, the way of consumer interaction has changed dramatically. From the earliest MicroBlogs, Little red book, and other online forums, to live streaming platforms such as Tik Tok, Kwai, and Taobao. Some of the network users complete the transformation from senior consumers to key opinion leaders, and then to streamers. The streamer is a content creator on a live shopping platform (Li, 2018) that attracts a large number of fans through e-commerce or online advertising (Marwick and Boyd, 2010), and converts online traffic into a sales force (Park and Lin, 2020). Streamers are often seen as experts capable of providing reliable information and practical buying advice (Chetioui et al., 2020). They need to not only clearly explain the features and functions of the products on sale (Hu et al., 2017), but also interact effectively with customers (Lim et al., 2020). Some successful streamers attract a large number of fans with their charisma, and original and high-quality content (Park and Lin, 2020). They even have more followers than traditional endorsers (Chen et al., 2016; Yu et al., 2016). The commercial value of streamers with large followings is also growing as the popularity of live shopping platforms increases (De Veirman et al., 2017; Hill et al., 2020).

In China, the two most well-known streamers, Li Jiaqi and Viya are affectionately called “close guy friends” and “female big brothers” by consumers. The close guy friend and female big brother are both distinctly androgynous, meaning they are both masculine and feminine, and this androgynous gender role is significantly different from traditional gender stereotypes. So, is it inevitable or accidental that the streamers Li Jiaqi and Viya, who are consumers’ “close guy friends” and “female big brothers,” are highly sought after by consumers for their androgynous characteristics? Are androgynous streamers more popular than traditional single-gender streamers? It is difficult for existing studies to provide insightful explanations for this question.

The personal attributes of the streamers are important factors affecting the live streaming effects (Todd and Melancon, 2018). Existing studies on the personal attributes of streamers have mostly focused on the ability of streamers to control time (Wang et al., 2021), the reliability of streamers, including motivation and moral quality (Chung and Cho, 2017), the interactivity between streamers and consumers (Sun et al., 2019), and the ability of streamers to evoke emotions in consumers (Xiao and Guo, 2020) and other factors on the influence of live streaming effects, but lack of research on the market effect of the androgynous streamers. Therefore, to remedy the limitations of previous studies, this research explores the influence of streamers’ gender roles (single gender/androgyny) on consumers’ product preferences according to attachment theory.

The current research consists of 2 studies: Study 1 verified the effect of streamers’ gender role on consumers’ product preferences. Androgynous streamers are more likely to promote consumers’ product preferences than single-gender streamers. Study 2 introduced individual gender stereotype level as a moderating variable and testified to the moderating effect of consumer gender stereotype level on the relationship between streamers’ gender roles and consumers’ product preferences in both female and male streamer contexts.

## Review and hypothesis development

### Streamer personal attributes

Streamers’ ability to control time is a key factor affecting the effect of live streaming. Streamers adopt “starvation marketing” in live-streaming to increase the time pressure of the purchase process (e.g., limited-time sales), thus increasing consumers’ perceptions of the scarcity and high value of the products, which guides them to make impulsive purchases decisions (Wang et al., 2021). Time pressure is an important factor influencing individuals’ consumption decisions. Streamers can offer certain price discounts for a limited time to drive consumers to make impulse purchases. Consumers in this context develop a strong sense of time urgency, which affects consumers’ ability to obtain complete information and evaluate products (Hu et al., 2017). At the same time, the shortened decision time leads to the suppression of consumers’ cognitive functions, resulting in a perceived opportunity cost of “not buying will be regret.” According to the deadline effect, the closer the promotion is to the end, the greater the sense of urgency consumers have to purchase. The high time pressure created by the streamers increases the consumers’ willingness to make a purchase decision (Hong, 2021).

Second, consumers’ perception of the reliability of the streamers is also a key factor affecting the live streaming effect. Positive moral qualities such as honesty and integrity of streamers reflect high reliability (Dholakia and Sternthal, 1977; Erdogan, 1999), contribute to improving brand credibility (Chung and Cho, 2017), and enhance the live streaming effect (Lafferty and Goldsmith, 1999). When users perceive the non-conformity between the product and the streamer, they consider the streamer as a commercially motivated marketer and reduce the perception of the streamer’s reliability, which leads to a negative attitude toward live streaming (Yoo and Jin, 2015).

The interactivity of the streamers is also an important factor in consumers’ product preferences. Real-time communication and interaction between consumers and streamers can increase familiarity, shorten psychological distance, and promote a close relationship (Gunawardena and Zittle, 1997). Streamers can provide personalized shopping guidance according to consumers’ emotional appeals during the interaction (Sun et al., 2019). Consumers can receive targeted product information and personalized live experiences by interacting with streamers in real time. In the process of interaction, consumers’ shopping experiences and social presence are correspondingly enhanced, which makes them feel relaxed and happy (Al-Emadi and Ben Yahia, 2020).

Finally, the emotional arousal of the streamer is also an important factor that affects the live streaming effect. During social interactions, an individual’s emotions can evoke similar emotional experiences in others (Weinberg and Gottwald, 1982). The positive emotions expressed by the streamer when recommending a product can evoke positive emotions in consumers. Using highly emotional expressions to recommend products in live streaming can generate the herding effect and consumer convergence effect. Streamers also can awaken consumers’ emotions through lotteries, countdown seconds, etc. In the interactive session, they mobilize consumers’ emotional experience by organizing bullet chatting, commenting, answering questions, giving gifts, and other ways. In addition, streamers also ask questions for consumers to answer and share their stories to awaken consumers’ emotional feelings (Xiao and Guo, 2020).

### Gender roles

Gender roles are profiles of an individual’s gender-type characteristics, attitudes, and interests (Garnets and Joseph-H, 1979). In contrast to biological sex, gender roles are considered psychological gender, which is gradually constructed by the process of socialization (Kaplan and Mary-Anne, 1980). Gilbert (1985) argues that gender roles refer to the normative expectations of gender division in social interaction that exist in specific historical or cultural contexts, emphasizing the important role of social factors. Gender roles reflect the behavioral patterns of individuals in a sociocultural context.

Initial research follows the dualism of gender roles, suggesting that gender roles encompass a single masculine or feminine dimension, and consequently produce evident gender stereotypes. Subsequent research argues that there are multiple dimensions of individual gender roles. In 1974, Bem introduces the concept of androgyny, which refers to individuals having both masculine and feminine dimensions (Bem, 1974). Bem (1974) classifies gender role combinations as masculine, feminine, androgyny, and gender undifferentiated according to the strength of the masculinity and femininity scores. Bem and Lewis (1975) argues that traditional gender dualism hinders the full development of the individual’s personality, and androgyny allows individuals to show greater adaptability in different situations. Qian et al. (2000) also classify college students in China into masculine, feminine, androgyny, and undifferentiated gender roles, and find that the proportion of androgyny was highest among college students. Subsequent studies have verified similar conclusions that the diversification of gender roles is prevalent in cross-cultural contexts (Spence, 1993).

With the interpenetration of male and female occupations, the boundaries of male and female gender roles are becoming blurred. Traditional gender stereotypes of both sexes are merging, androgyny has become a trend. The proposal of androgyny means that individuals are free to express masculine or feminine characteristics at the same time, without following the traditional gender stereotypes. Bem (1974) argues that androgyny implies a more flexible and healthy psychological state. Spence (1993) show that androgynous individuals are more malleable and adaptive. Androgynous children are more assertive, socially responsible, and competent. Boys are more creative, and girls have more active thinking and stronger leadership skills. Androgynous adolescents have higher levels of mental health and self-esteem, more positive self-evaluations, etc. They have more confidence and higher competence (Cheng, 2005).

The positive impact of androgyny is not limited to the individual who possesses it; it also affects how others perceive this person. Jackson (1983) argues that gender role has a significant impact on human perception. Androgynous individuals are perceived by others as lovable and attractive, and thus more likely to succeed in their careers. Lieven and Hildebrand (2016) discovers that the success of Jimi Hendrix, the Beatles, and Elvis Presley is not only due to their musical ability, but also their tendency to position themselves as androgynous rather than masculine or feminine, ultimately appealing to both women and men. Androgyny also affects employees’ perceptions of leaders. Kark et al. (2012) shows that female leaders will pay a higher penalty than male leaders when they are not “androgynous,” as employees are less likely to identify with them. In interpersonal relationships, androgynous people also give others a better interaction experience than traditional gender roles. Hirokawa et al. (2000) argues that androgynous individuals have more abundant conversation, which makes others feel more approachable, and thus have better interpersonal relationships. Izod (1995) also finds androgynous people are more popular with young people than traditional gender roles.

### Attachment theory

Attachment theory is firstly introduced by John Bowlby (1969). Attachment refers to the long-term emotional bond formed between an individual (mainly infants) and another individual (mother or primary nurturer). Ainsworth et al. (1978) inherits and develops attachment theory by applying the strange situation to measure children’s attachment behaviors toward their mothers. According to the experimental results, he divides the attachment types into secure, anxious, and avoidant. Shaver and R-Chris (2004) extend attachment theory to the adult domain. They discover that adult relationships are also a form of attachment, which can be classified as secure, anxious, and avoidant as well.

Attachment can significantly influence an individual’s behavior and motivation. Paulssen and Fournier (2007) argue that attachment has applicability in different relationship situations. People also have attachment patterns in the field of business relationships, such as consumers’ attachment to celebrity endorsers. Ilicic and Webster (2011) show that attachment to endorsers promotes consumers to have a more positive attitude and brand preference. Ilicic et al. (2016) find that consumers’ need for autonomy and connection can significantly influence consumers’ brand attachment. Saldanha et al. (2020) illustrate that when the attachment between consumers and brand spokesman is higher, consumers are more likely to purchase the product. Subsequent research also reveals the influence of different attachment styles on individual preferences. Thomson and Johnson (2006) distinguish two types of consumer attachment, anxious and avoidant, and demonstrate that consumers can satisfy their missing interpersonal needs and have more positive emotional experiences in a business relationship through attachment to brands. Jeong and Drolet (2010) examine the influence of attachment type on individuals’ preferences and find that individuals with high attachment anxiety hold more positive attitudes toward partner-focused ads than self-focused ads.

Adams-Price and Greene (1990) classified attachment into two types: Identity attachment and romantic attachment, based on the social goals of attachment. This study argues that individuals’ attachment to celebrities refers to the feeling or imaginary relationships characterized by an individual’s understanding of distant objects. Attachment can significantly contribute to the development of an individual’s social identity and gender identity. Identity attachment is more likely to occur between people of the same sex, and it stems from the individual’s internal need for self-actualization. It means that an individual identifies with others’ attributes, such as personality, ability, or authority, and wants to acquire these characteristics, which can promote the development of the individual’s social identity. Romantic attachment is more likely to occur between heterosexuals and stems from the psychological need to pursue ideal romantic relationships, meaning that individuals admire the attachment partner, want to become the attachment partner’s lover and establish the ideal romantic relationship, which can promote the development of individual gender identity (Adams-Price and Greene, 1990). Yue (1999) shows that women rely more on romantic attachment. The worship of idols is mainly romantic attachment, and they desire to establish romantic relationships with their idols. Men rely more on identity attachment. The worship of idols is mainly identity attachment, and they appreciate the professional ability of their idols.

### The influence of streamers’ gender role (single gender/androgyny) on consumers’ preferences

This study concludes that the streamers’ gender role significantly influences consumers’ preferences. Specifically, when the streamers are single gender, they are more likely to influence consumers’ preferences through a single identity attachment (same-sex consumers) or a single romantic attachment (heterosexual consumers). However, when the streamers are androgynous, they are more likely to influence consumers’ preferences through both identity attachment and romantic attachment.

When the streamers are single gender, same-sex consumers are more likely to recognize their professional ability and develop an identity attachment. For example, the effect of female endorsers on the endorsement of female products is better, and this effect also appears in the male endorsers’ conditions (Zhang and Sun, 2019), because same-sex endorsers are more professional and representative of same-sex products. In the sales environment, female consumers perceive female sales as more professional in selling female products and therefore identify more with female sales (Stros et al., 2018). Male also admire same-sex celebrities more, see same-sex celebrities as role models, and tend to purchase brands endorsed by role models (Yue, 1999). Escalas and Bettman (2017) argue that consumers identify the products endorsed by same-sex endorsers as a way to construct their self-concept which subsequently increases their purchase intentions. Consumers’ identity attachment in the process of identifying with the professionalism of same-sex streamers promotes positive attitudes toward the products. Therefore, same-sex consumers tend to activate their identity attachment and increase positive attitudes toward products when the streamers are single gender.

When the streamers are single gender, heterosexual consumers are more inclined to initiate a romantic attachment which increases their positive attitude toward live products. Existing research shows that fans are more likely to worship idols of the opposite sex and express their love for idols by spending money to support them. Fans often refer to themselves as “boy/girlfriend” and “husband/wife” and develop a romantic attachment to their idols, hoping to establish a romantic relationship with their idols (Yue and Yan, 2007). Consumers also spend money on heterosexual idols in exchange for the opportunity to contact them, and purchase brands associated with idols to draw closer to them (Cheah et al., 2019; Park et al., 2019). Therefore, consumers’ romantic attachment to heterosexual streamers raises their product preferences. As a result, heterosexual consumers tend to activate their romantic attachment and increase their positive attitudes toward live products when the streamers are single gender.

When the streamers are androgynous, they have both masculine and feminine qualities. For consumers, the androgynous streamer can activate both the professional advantage brought by gender consistency and the romantic relationship tendency initiated by heterosexual charm. Therefore, compared to the single-gender streamers who influence consumers through a single identity attachment or romantic attachment, the androgynous streamers can activate both romantic attachment and identity attachment of consumers, which can effectively increase consumers’ positive attitudes toward live products. Because androgynous streamers have both the professional advantage and the heterosexual charm, they are more persuasive and attractive to consumers than single-gender streamers. Therefore, compared with single-gender streamers, androgynous streamers increase consumers’ positive attitudes toward live products.

H1: Androgynous streamers can elicit higher consumer preferences toward live-streaming products than single-gender streamers.

### The moderating role of gender stereotypes

Stereotypes are categorized according to gender, race or occupation, etc., and eventually form a fixed impression about a certain type of group. Gender stereotypes are one of the important components of stereotypes, which refer to the relatively fixed views and perceptions of male and female gender attribute that are widely accepted in social life (Goodwin et al., 2000). For example, men are perceived as competitive, assertive, and dominant, while women are perceived as gentle, kind, and submissive. When individuals have a high level of gender stereotypes, they are more likely to endorse gender role information that is consistent with gender stereotypes and ignore gender role information that is inconsistent with gender stereotypes (Goodwin et al., 2000).

Gender stereotypes are important factors to influence consumers’ preferences. Research shows that female cues are associated with the perceptions of brand warmth, male cues are associated with the perceptions of brand competence, and the sense of brand warmth and competence contributes to consumers’ preferences (Aaker et al., 2012; Kervyn et al., 2012). Brough et al. (2016) show that sustainable consumption is strongly associated with femininity and female gender stereotypes, so men reduce green consumption to maintain their gender identity. Luoh and Sheng-Hshiung (2007) examine the influence of consumers’ gender stereotypes on the perceptions of service quality. Consumers with high levels of gender stereotypes report a higher service quality and more willingness to pay for female waiters who conform to gender stereotypes (Luoh and Sheng-Hshiung, 2007).

In this study context, the level of consumers’ gender stereotypes can effectively influence the relationship between the streamers’ gender roles and consumers’ product preferences. Specifically, when consumers have high levels of gender stereotypes, single-gender streamers promote more positive consumer attitudes toward products than androgynous streamers.

Consumers with high levels of gender stereotypes pay more attention to the messages that are consistent with gender stereotypes and ignore the messages that are inconsistent with gender stereotypes. The single-gender streamers conform to traditional gender stereotypes and meet the psychological expectations of consumers with high levels of gender stereotypes, so consumers will be more likely to pay attention to the information related to the single-gender streamers, which increases their preferences for the live products. However, androgynous streamers do not conform to traditional gender stereotypes and the psychological expectations of consumers with high gender stereotypes. Therefore, consumers are less inclined to endorse the information related to androgynous streamers, which reduces their preferences for live products. As a result, when consumers have high levels of gender stereotypes, the single-gender streamers can promote more positive attitudes of consumers toward the products than the androgynous streamers.

When consumers have low levels of gender stereotypes, androgynous streamers promote more positive attitudes toward live products than single-gender streamers. Consumers with low levels of gender stereotypes are less likely to be influenced by gender stereotypes and have less fixed expectations about the streamers’ gender roles. At this point, the single-gender streamers influence consumers’ positive attitudes by activating a single identity attachment or romantic attachment, while the androgynous streamers can activate consumers’ identity attachment and romantic attachment at the same time, which increases consumers’ positive attitudes toward live products. Therefore, when consumers have a low level of stereotypes, androgynous streamers are more likely to promote consumers’ preferences than single-gender streamers.

H2: The level of consumers’ gender stereotypes can significantly moderate the relationship between the streamers’ gender roles and consumers’ product preferences. When consumers have a high level of gender stereotypes, single-gender streamers elicit higher consumer preferences than androgynous streamers. When consumers have a low level of gender stereotypes, androgynous streamers promote a more positive consumer attitude than single-gender streamers.

## Materials and methods

### Study 1

Study 1 tested the basic premise that the gender role of streamers (single gender/androgyny) could significantly affect consumers’ preferences, which supported H1.

#### Participants and design

Study 1 recruited 210 participants from a public university in China to complete a series of experiments. The participants were randomly assigned to a 2 (streamer: male/female) × 2(single gender/androgyny) experimental design. The final sample size was 187 (female 51.34%, age from 18 to 33, *M* = 22.68, SD = 3.02; *n*_*male–single*_ = 47, *n*_*male–and*_ = 46, *n*_*female–single*_ = 48, *n*_*female–and*_ = 46).

#### Stimuli and procedure

The researchers employed an electric toothbrush as the product in live broadcast, which did not exist the gender orientation (Stålnacke et al., 1995). Live videos with four versions (masculine, androgynous male, feminine, androgynous female) were made as stimulus materials (see Appendix for details). The masculine male streamer and the androgynous male streamer were played by the same man, and the feminine female streamer and androgynous female streamer were also played by the same woman. The researchers controlled for other factors including product, price, lighting effects, equipment, background environment, and other variables. To ensure that the manipulation of the gender role of streamer in study 1 was efficient, an initial group of participants from the internet (*n* = 132, female 53.03%, age from 18 to 30, *M* = 22.26, SD = 2.39) was recruited to participate into a pretest. All the participants were randomly assigned to a 2(streamer: male/female) × 2(single gender/androgyny) experimental design.

Each group was asked to watch the relevant live video, and then completed the gender role scale (BSRI Bem, 1974; 7-point scale, 1 = completely disagree and 7 = completely agree) on the streamer. When the average score was greater than 4 in the male items, and less than 4 in the female items, the streamer was masculine. When the average score was greater than 4 in the female items, and less than 4 in the male items, the streamer was feminine. When the average scores in both male and female items were greater than 4, the streamer was androgyny.

The results revealed that the masculine group reported an average score higher than 4 in male items, and less than 4 in female items (*M*_*male*_ = 4.55, SD = 0.97, *M*_*female*_ = 2.21, SD = 0.89, *t* = 9.84, df = 32, *p* < 0.05). The androgynous male group reported average scores higher than 4 both in the male items and female items (*M*_*male*_ = 4.39, SD = 0.97, *M*_*female*_ = 4.27, SD = 0.98, *t* = 0.47, df = 32, *p* > 0.05). The feminine group reported an average score higher than 4 in female items, and less than 4 in male items (*M*_*male*_ = 2.36, SD = 0.90, *M*_*female*_ = 4.48, SD = 0.87, *t* = 10.70, df = 32, *p* < 0.05). The androgynous female group also reported average scores higher than 4 both in the male items and female items (*M*_*male*_ = 4.27, SD = 0.94, *M*_*female*_ = 4.39, SD = 1.03, *t* = 0.51, df = 32, *p* > 0.05). The findings verified the viability of the manipulation of streamers’ gender roles in study 1.

In the main experiment, the researchers told the participants the purpose of this activity was to test a new product. The participants were randomly assigned to a 2(streamer: male/female) × 2(single gender/androgyny) experimental design. Each group watched the relevant live video and then completed a series of surveys, including the gender role scale on the streamer (Bem, 1974), and reported their preferences for the product (Lee and Pillai, 2013; 7-point scale, 1 = dislike, 7 = like). The key questions appeared interspersed with irrelevant items about the impression of the researcher, individual interests, and activity suggestions, among others. To rule out the influence of the streamer’s language style (Berryman-Fink and Wilcox, 1983) and professionalism (Ohanian, 1990), participants also reported the language style and professionalism of the streamer. Finally, they reported whether their attitudes to the product were based on their past shopping experiences and guessed the purpose of this experiment.

#### Results and discussion

*Manipulation check:* 16 participants’ attitudes depended on past shopping experiences, and 7 participants guessed the real purpose of the experiment. The results revealed that the masculine group reported an average score higher than 4 in male items, and less than 4 in female items (*M*_*male*_ = 4.49, SD = 0.93, *M*_*female*_ = 2.53, SD = 0.95, *t* = 11.57, df = 46, *p* < 0.05). The androgynous male group reported average scores higher than 4 both in the male items and female items (*M*_*male*_ = 4.35, SD = 0.92, *M*_*female*_ = 4.26, SD = 0.88, *t* = 0.54, df = 45, *p* > 0.05). The feminine group reported an average score higher than 4 in female items, and less than 4 in male items (*M*_*male*_ = 2.40, SD = 0.92, *M*_*female*_ = 4.63, SD = 0.87, *t* = 11.95, df = 47, *p* < 0.05). The androgynous female group also reported average scores higher than 4 both in the male items and female items (*M*_*male*_ = 4.24, SD = 0.97, *M*_*female*_ = 4.41, SD = 0.93, *t* = 0.84, df = 45, *p* > 0.05). No matter whether the streamer was male or female, there was no significant difference on the streamer’s language style (*M*_*single*–male_ = 4.21, SD = 0.78, *M*_*and*–male_ = 4.37, SD = 0.71, *t* = 1.01, df = 91, *p* > 0.05; *M*_*single*–female_ = 4.46, SD = 0.85, *M*_*and*–female_ = 4.59, SD = 0.88, *t* = 0.51, df = 92, *p* > 0.05) and professionalism (*M*_*single*–male_ = 4.13, SD = 0.90, *M*_*and*–male_ = 4.02, SD = 0.91, *t* = 0.57, df = 91, *p* > 0.05; *M*_*single*–female_ = 4.25, SD = 1.10, *M*_*and*–female_ = 4.17, SD = 0.97, *t* = 0.35, df = 92, *p* > 0.05) between the two groups. The results showed that the experiment manipulation effectively influenced the majority of participants.

*Product preference:* The results discovered that the gender role of the streamer could significantly influence consumers’ product preferences. When the streamer was male, participants in the androgyny group report significantly higher product preferences than those in the single-gender group (*M*_*and*_ = 4.43, SD = 1.22, *M*_*single*_ = 3.70, SD = 1.18, *t* = 2.94, df = 91, *p* < 0.05). When the streamer was female, participants in the androgyny group also reported significantly higher product preferences than those in the single-gender group (*M*_*and*_ = 4.37, SD = 1.08, *M*_*single*_ = 3.90, SD = 1.15, *t* = 2.05, df = 92, *p* < 0.05).

*Multi-mediation analysis*: To rule out the influence of streamer’s language style and professionalism, the researchers used SPSS Macro software proposed by (Preacher and Hayes, 2008), with the gender role of streamers as the independent variable, consumers’ preferences as the dependent variable, language style and professionalism as the mediator variables for multiple mediation analysis.

The results showed that the gender role of streamers did not affect the language style and professionalism in both male streamer (β_*languagestyle*_ = −0.16, CI95: low = −0.46; high = 0.15; β_*professionalism*_ = 0.12, CI95: low = −0.26; high = 0.49) and female streamer (β_*languagestyle*_ = −0.13, CI95: low = −0.48; high = 0.23; β_*professionalism*_ = 0.05, CI95: low = −0.37; high = 0.47) condition. The language style and professionalism were not able to influence the consumers’ preferences in both male streamer (β_*languagestyle*_ = 0.12, CI95: low = −0.21; high = 0.46; β_*professionalism*_ = 0.11, CI95: low = −0.17; high = 0.39) and female streamer (β_*languagestyle*_ = −0.06, CI95: low = −0.33; high = 0.21; β_*professionalism*_ = 0.12, CI95: low = −0.11; high = 0.34) condition. However, the language style and professionalism did not mediate the relationship between the gender role of streamers and consumers’ preferences in both male streamer (β_*languagestyle*_ = −0.02, CI95: low = −0.20; high = 0.03; β_*professionalism*_ = 0.01, CI95: low = −0.03; high = 0.14) and female streamer (β_*languagestyle*_ = 0.01, CI95: low = −0.03; high = 0.13; β_*professionalism*_ = 0.01, CI95: low = −0.05; high = 0.11) condition.

Study 1 showed that the gender role of streamers could significantly affect consumers’ product preferences. Regardless of whether the streamer was male or female, the androgynous streamer could elicit a higher product preference than the single-gender streamer, which verified H1.

### Study 2

Study 2 included 2 sub-studies, which testified to the moderating effect of individuals’ gender stereotype level on the relationship between streamers’ gender roles and consumers’ preferences in both male and female stream conditions.

#### Study 2a

Study 2a explored the moderating role of individuals’ gender stereotype level in the relationship between female stream genders’ roles and consumers’ preferences.

*Participants and Design*: Study 2a recruited 210 participants from a public university in China to complete a series of experiments. The participants were randomly assigned to a 2(gender stereotype: high/low) × 2(female: feminine/androgyny) experimental design. The final sample size was 185 (female 48.11%, age from 18 to 32, *M* = 22.94, SD = 3.02; *n*_*female–and*–high_ = 48, *n*_*female–and*–low_ = 44, *n*_*female–single*–high_ = 45, *n*_*female–single*–low_ = 48).

*Stimuli and Procedure:* The researchers employed a jewelry necklace as the live broadcast product. Live videos with two versions (feminine/androgyny) were made as stimulus materials (see Appendix for details), and the feminine female streamer and androgynous female streamer were played by the same people. The researchers controlled for other factors including product, price, lighting effects, equipment, background environment, and other variables. To ensure that the manipulation of the gender role of streamer in study 2a was successful, an initial group of participants from the internet (*n* = 83, female 53.01%, age from 18 to 26, *M* = 21.13, SD = 1.92) was recruited to participate into a pretest. All the participants were randomly assigned to two groups (feminine/androgyny).

All the participants were asked to watch the relevant live video and then completed the gender role scale on the streamer (Bem, 1974), and reported the language style and professionalism of the streamer. The findings revealed that the feminine group reported an average score higher than 4 in female items, and less than 4 in male items (*M*_*male*_ = 2.54, SD = 0.92, *M*_*female*_ = 4.49, SD = 1.00, *t* = 8.84, df = 40, *p* < 0.05). The androgyny group reported average scores higher than 4 both in the male items and female items (*M*_*male*_ = 4.21, SD = 1.02, *M*_*female*_ = 4.55, SD = 0.92, *t* = 1.74, df = 41, *p* > 0.05). There was no significant difference on the streamer’s language style (*M*_*single*–female_ = 4.34, SD = 0.82, *M*_*and*–female_ = 4.36, SD = 0.85, *t* = 0.09, df = 81, *p* > 0.05) and professionalism (*M*_*single*–female_ = 4.12, SD = 0.84, *M*_*and*–female_ = 4.05, SD = 0.76, *t* = 0.42, df = 81, *p* > 0.05) between the two groups.

The findings showed the viability of the gender role employed in study 2a.

In the main experiment, the researchers told the participants the purpose of this activity was to test a new product. The participants were randomly assigned to two groups (female: feminine/androgyny). The participants were asked to watch the relevant live video, and then complete a series of surveys, including the gender role stereotypes scale (Mills et al., 2012), the gender role of the streamer, and the preference for the product. The key questions appeared interspersed with irrelevant items about the impression of the researcher, individual interests, and activity suggestions, among others. Finally, they reported whether their attitudes to the product were based on their past shopping experiences and guessed the purpose of this experiment.

*Manipulation check:* 20 participants’ attitudes depended on past shopping experiences, and 5 participants guessed the real purpose of the experiment. The results revealed that the feminine group reported an average score higher than 4 in female items, and less than 4 in male items (*M*_*male*_ = 2.63, SD = 0.99, *M*_*female*_ = 4.77, SD = 0.84, *t* = 18.13, df = 92, *p* < 0.05). The androgyny group reported average scores higher than 4 both in the male items and female items (*M*_*male*_ = 4.33, SD = 0.96, *M*_*female*_ = 4.58, SD = 1.07, *t* = 1.68, df = 91, *p* > 0.05). The results showed that the experiment manipulation effectively influenced the majority of participants.

*Product preference:* The findings revealed a significant interaction of individuals’ gender stereotype level and streamers’ gender roles on product preferences in the female stream situation (*F* = 19.30, *p* < 0.001). The simple slope test showed that when individuals had a high gender stereotype (one SD higher than the average), the gender role of streamer significantly affected product preference (*B* = −2.07, *t* = −18.92, *p* < 0.001, 95% CI = [lower bound -2.29, upper bound -1.86]). The feminine streamer could elicit a higher product preference than the androgyny streamer (*M*_*androgyny*_ = 2.44, SD = 0.62, *M*_*feminine*_ = 5.17, SD = 0.38, *t* = 15.92, df = 34, *p* < 0.05). For individuals with a low gender stereotype (one SD lower than the average), the gender role also can significantly influence product preference (*B* = 1.18, *t* = 10.72, *p* < 0.001, 95% CI = [lower bound 0.96, upper bound 1.39]). In this condition, the androgyny streamer could elicit a higher product preference than the feminine streamer (*M*_*androgyny*_ = 5.19, SD = 0.54, *M*_*feminine*_ = 2.94, SD = 0.44, *t* = 12.84, df = 30, *p* < 0.05).

Study 2a showed that individuals’ gender stereotype levels moderated the relationship between streamers’ gender roles and consumers’ preferences in the female streamer condition. when individuals had a high gender stereotype, the feminine streamer could lead to a higher product preference than the androgyny streamer. when individuals had a low gender stereotype, the androgyny streamer could promote a higher product preference than the feminine streamer.

#### Study 2b

Study 2b verified the moderating effect of individuals’ gender stereotype level on the relationship between male streamers’ gender roles and consumers’ preferences.

*Participants and Design*: Study 2b recruited 210 participants from a public university in China to complete a series of experiments. The participants were randomly assigned to a 2(gender stereotype: high/low) × 2(male: masculine/androgyny) experimental design. The final sample size was 182 (female 43.96%, age from 18 to 28, *M* = 22.10, SD = 2.17; *n*_*male–and*–high_ = 44, *n*_*male–and*–low_ = 47, *n*_*male–single*–high_ = 46, *n*_*male–single*–low_ = 45).

*Stimuli and Procedure:* The researchers employed a wristwatch as the live broadcast product. Live videos with two versions (male: masculine/androgyny) were made as stimulus materials (see Appendix for details), and the masculine male streamer and the androgynous male streamer in the live broadcast were played by the same people. The researchers controlled for other factors including product, price, lighting effects, equipment, background environment, and other variables. To ensure that the manipulation of the gender role of streamers in study 2b was successful, an initial group of participants from the internet (*n* = 85, female 45.88%, age from 18 to 27, *M* = 21.34, SD = 1.86) was recruited to participate into a pretest. All the participants were randomly assigned to two groups (male: masculine/androgyny).

All the participants were asked to watch the relevant live video and then completed the gender role scale on the streamer (Bem, 1974), and reported the language style and professionalism of the streamer. The findings revealed that the masculine group reported an average score higher than 4 in male items, and less than 4 in female items (*M*_*male*_ = 4.58, SD = 1.12, *M*_*female*_ = 2.26, SD = 0.90, *t* = 10.01, df = 42, *p* < 0.05). The androgyny group reported average scores higher than four both in the male items and female items (*M*_*male*_ = 4.45, SD = 1.02, *M*_*female*_ = 4.33, SD = 0.85, *t* = 0.68, df = 41, *p* > 0.05). There was no significant difference on the streamer’s language style (*M*_*single*–male_ = 4.33, SD = 0.84, *M*_*and*–male_ = 4.26, SD = 0.77, *t* = 0.37, df = 83, *p* > 0.05) and professionalism (*M*_*single*–male_ = 4.07, SD = 0.86, *M*_*and*–male_ = 3.95, SD = 0.73, *t* = 0.68, df = 83, *p* > 0.05) between the two groups. The findings showed the viability of the gender role employed in study 2b.

In the main experiment, the researchers told the participants the purpose of this activity was to test a new product. The participants in each group were asked to watch the relevant live video, and then complete a series of surveys (the same as study 2a), including the gender role stereotypes scale, the gender role of the streamer, and the preference for the product. The key questions appeared interspersed with irrelevant items about the impression of the researcher, individual interests, and activity suggestions, among others. Finally, they reported whether their attitudes to the product were based on their past shopping experiences and guessed the purpose of this experiment.

*Manipulation check:* 21 participants’ attitudes depended on past shopping experiences, and 7 participants guessed the real purpose of the experiment. The results revealed that the masculine group reported an average score higher than 4 in male items, and less than 4 in female items (*M*_*male*_ = 4.85, SD = 1.04, *M*_*female*_ = 2.82, SD = 1.01, *t* = 12.28, df = 90, *p* < 0.05). The androgyny group reported average scores higher than 4 both in the male items and female items (*M*_*male*_ = 4.59, SD = 0.94, *M*_*female*_ = 4.24, SD = 1.06, *t* = 2.27, df = 90, *p* < 0.05). The results showed that the experiment manipulation effectively influenced the majority of participants.

*Product preference:* The results showed a significant interaction of individuals’ gender stereotype level and male streamers’ gender roles on product preferences (*F* = 41.51, *p* < 0.05). The simple slope test showed that when individuals had a high gender stereotype (one SD higher than the average), the gender role of the streamer significantly affected product preference (*B* = −1.15, *t* = −7.64, *p* < 0.001, 95% CI = [lower bound -1.45, upper bound -0.85]). The masculine streamer could elicit a higher product preference than the androgyny streamer (*M*_*androgyny*_ = 2.89, SD = 0.32, *M*_*masculine*_ = 5.14, SD = 0.36, *t* = 20.96, df = 38, *p* < 0.05). For individuals with a low gender stereotype (one SD lower than the average), the gender role also can significantly influence product preference (*B* = 0.70, *t* = 4.64, *p* < 0.001, 95% CI = [lower bound 0.40, upper bound 1.00]). In this condition, the androgyny streamer could lead to a higher product preference than the masculine streamer (*M*_*androgyny*_ = 3.38, SD = 0.50, *M*_*masculine*_ = 2.94, SD = 0.25, *t* = 3.13, df = 30, *p* < 0.05).

Study 2b testified to the moderating role of individuals’ gender stereotype level in the relationship between streamers’ gender roles and consumers’ preferences in the male streamer condition, which supported H2.

## General discussion

### Conclusion

Study 1 showed that the gender role of streamers (single gender/androgyny) could significantly affect consumers’ product preferences. Regardless of whether the streamer was male or female, the androgynous streamer could elicit a higher product preference than the single-gender streamer. Study 2 employed individuals’ gender stereotype level as moderator, and explored its’ moderating role in the relationship between stream gender role and consumer preference both in the female and male streamer condition. The findings discovered when individuals had a high gender stereotype, the single-gender streamer could lead to a higher product preference than the androgynous streamer. When individuals had a low gender stereotype, the androgynous streamer could promote a higher product preference than the single-gender streamer.

### Research contributions

Firstly, previous studies mainly focus on streamers’ ability to control time (Wang et al., 2021) and arouse consumers’ emotions (Xiao and Guo, 2020), the reliability of streamers, including motivations and moral quality (Chung and Cho, 2017), the interaction between streamers and consumers (Sun et al., 2019), etc. The existing studies do not explore the impact of streamers’ gender roles on consumers’ preferences in the context of live broadcasting. Therefore, the current research first introduces the two-dimensional variables of streamer gender role (single gender/androgyny), which provides a new research perspective for explaining the influence of streamers’ gender roles on the live-broadcast effect. This study expands the research on gender roles in the field of live broadcasts. The existing studies on gender role type mostly explore the influence of gender roles on individual growth, mental health, social adaptation, and other fields (Spence, 1993; Cheng, 2005). Based on the previous studies, this manuscript makes a comparative analysis of the two types of gender roles (single gender/androgyny) and discusses their impact on consumer product preferences. Under the new background of the network broadcast, this study further analyzes the influence of streamers’ gender roles on consumer behavior, enriches the application perspective of gender role theory, and opens up a new direction for subsequent research.

Secondly, based on the attachment theory, this study analyzes the influence of streamers’ gender roles on consumers’ preferences. Previous studies on attachment theory mainly focus on attachment style, interpersonal relationships, advertising, and other fields (Paulssen and Fournier, 2007; Jeong and Drolet, 2010; Ilicic and Webster, 2011), which do not analyze the role of attachment between streamers and consumers. This study focuses on the specific situation of the network broadcast and explores the influence of streamer gender role on consumers’ preferences through two different types of attachment (identity attachment and romantic attachment). This study identifies the theoretical connection between streamer gender role and consumers’ attachment type, thus making an in-depth theoretical exploration of how streamer gender role affects consumers’ preferences, which makes up for the limitations in the existing research.

Finally, this study also explores the moderating effect of gender stereotype level and establishes boundary conditions for the main effect. Existing studies mainly focus on green consumption (Brough et al., 2016), service quality (Luoh and Sheng-Hshiung, 2007), gender cues, and brand perception (Aaker et al., 2012; Kervyn et al., 2012), etc. Few studies have examined the impact of gender stereotype levels on consumer decision-making in the context of the live broadcast. The current research first introduces the gender stereotype level into the main research framework and confirms that when individuals had a high gender stereotype, the single-gender streamer could lead to a higher product preference than the androgynous streamer. When individuals had a low gender stereotype, the androgynous streamer could promote a higher product preference than the single-gender streamer. This study further expands the research context of gender stereotype level, expounds on the boundary conditions of the relationship between streamers’ gender roles and consumers’ preferences, and provides a new perspective in the theoretical and application fields.

### Future study

This study firstly explains the impact of streamers’ gender roles on consumers’ preferences from the perspective of attachment theory. Future research can analyze the live-broadcast effects and internal mechanisms of streamers’ gender roles from other theoretical perspectives (single gender/androgyny). In addition, consumers often associate purchase behavior with positive psychological experiences, while this study only focuses on the influence of streamers’ gender roles on consumers’ preferences. Future research can test the influence of streamers’ gender roles on consumers’ psychological experiences in a further step, such as likability, interactivity, and emotional arousal. The current research only tests the influence of androgynous gender roles on consumers’ product preferences in the online streamer context. Future research can further explore whether androgynous gender role elicits some negative effect in other contexts. Finally, the current study only explores the moderating effect of gender stereotype level on the relationship between streamers’ gender roles and consumers’ preferences. Future research can consider the moderating role of other variables, such as individual power motivation, and identity centrality.

## Data availability statement

The original contributions presented in this study are included in the article/Supplementary material, further inquiries can be directed to the corresponding author.

## Ethics statement

The studies involving human participants were reviewed and approved by the Ethics Committee of Research Center for Psychological and Health Sciences, China University of Geosciences. The patients/participants provided their written informed consent to participate in this study.

## Author contributions

FW was responsible for the logical reasoning of the research topic. XS and YY were responsible for experimental materials and data. HH was responsible for collecting literature. All authors contributed to the article and approved the submitted version.
