# Supplementary material for: The influence of androgynous streamers on consumers’ product preferences
Source: Front Psychol. 2022 Dec 1;13:1029503. doi: 10.3389/fpsyg.2022.1029503 (PMC9752884; doi:10.3389/fpsyg.2022.1029503)
Supplement: Supplementary file 1 [file Presentation_1.pdf]

Appendix

Study 1

Hello, everyone! Today I'm going to bring you an electric toothbrush which is very popular recently. This electric toothbrush is a new product designed by Brush brand for young people this year. First of all, the electric toothbrush uses the latest technology to vibrate with sound waves up to 30,000 times per minute. It improves the cleanliness of teeth and ensures that you can eat anything you want. Secondly, this electric toothbrush is designed with three models, clear, sensitive, and white. The clean model can effectively remove plaque! The sensitive mode is suitable for babies with sensitive teeth or porcelain teeth. I especially like the white model. You can see the whitening effect after four weeks of continuous use. My darling, buy now and get 50% off, only 299 yuan! Let's get started!

Androgynous male: accentuating gentle, light-colored clothing, and a slightly high-pitched male voice.

Androgynous female: highlight calm and competent characteristics, dark suits, and a low-pitched female voice.

Hi guys, today I bring you an electric toothbrush of Brush brand for young people this year. This toothbrush uses the latest technology of acoustic vibration, up to 30,000 times per minute. It is especially good at cleaning teeth and ensures that you can eat anything you want. It has three models: clean, sensitive, and white. The clean model can effectively remove dental plaque, and the sensitive model is suitable for those with sensitive teeth and porcelain teeth. The Whitening model function is very prominent. You can see the obvious whitening effect after continuous use for four weeks, which makes you smile with confidence. Buy now and get 50% off, only 299 yuan! Let's get started!

Masculine male: emphasis on masculinity, dark suit, and masculine bass

Hello, my darling! Today I bring you an electric toothbrush of Brush brand for young people this year. This toothbrush uses the latest technology of acoustic vibration, which vibrates up to 30,000 times per minute. It is excellent for cleaning teeth and making your teeth look beautiful. It has three models: clean, sensitive, and white. The clean model can effectively remove the black and dark things on your teeth! The sensitive model is suitable for babies with sensitive teeth or porcelain teeth. My favorite white model is very effective. You can see the whitening effect after four weeks of continuous use. My darling, buy now you can get a 50 % discount, only 299 yuan! Hurry up and take it home!

Feminine female: Highlight the female gentle, choose a skirt, long hair, girly eyeshadow, and feminine high notes

Study 2

Study 2a

Hello, everyone! Today I bring you a tourmaline necklace which is very popular recently. This necklace is fashion jewelry designed by Brush brand for young people this year. This necklace is made of 18-k gold and high-quality watermelon tourmaline from Brazil. Secondly, this necklace is very fashionable! According to the young people's favorite fruit elements, it makes you very pretty. Everybody, buy now, you can get a 70% discount, only 899 yuan! Let's take it home!

Androgynous female: highlight calm and competent characteristics, dark suits, and a low-pitched female voice.

Hello, my darling, today I bring you a very popular tourmaline necklace promoted by Brush brand this year. This necklace is specially designed for our beautiful young guys. It is made of 18-k gold with high-quality Brazilian watermelon tourmaline. This necklace is very stylish! The fruit element makes you so beautiful. My darling, buy now, you can get a 70% discount, only 899 yuan! Let's take it home!

Feminine female: Highlight the female gentle, choose a skirt, long hair, girly eyeshadow, and feminine high notes

Study 2b

Hello, everyone! Today I bring you a very popular wristwatch recently. This watch is very fashion designed by Brush brand for young people this year. It uses the latest alloy technology to prevent shock, water, and moisture. Secondly, this watch employs the sports style favored by young people. The appearance is very cool, which makes you full of confidence. Everybody, buy now, you can get a 70% discount, only need 899 yuan! Let's take it home!

Androgynous male: accentuating gentle, light-colored clothing, and a slightly high-pitched male voice.

Hello, guys! Today I bring you a popular wristwatch of Brush brand designed for young people this year. This watch uses the latest alloy technology to prevent shock, water, and moisture. Secondly, this watch has a strong sense of fashion! According to young people's favorite sports style, it looks so cool! Wearing this wristwatch can make you smile with confidence! Guys buy now, you can get a 70% discount, only need 899 yuan! Let's take action!

Masculine male: emphasis on masculinity, dark suit, and masculine bass

The Bem Sex-Role Inventory (BSRI; Bem, 1974)
Please read the following 40 items according to what you see in the live broadcast and evaluate the streamer from 1 to 7, where 1 = completely disagree and 7 = completely agree.

Female items
Warm
Gentle
Affectionate
Sympathetic
Sensitive to other’s needs
Tender

Male items
Has leadership abilities
Strong personality
Acts as leader
Dominant
Defends own beliefs
Makes decisions easily

Gender Role Stereotypes Scale
(Mills et al., 2021)

Please indicate the extent to which you believe each task should be done by the man (or by the woman).
7-point scale, 1 totally disagree, 7 totally agree

1 Mow the lawn
2 Drive the car, when both the man and the woman are traveling
3 Prepare meals
4 Propose marriage
5 Perform basic maintenance of vehicles, such as changing the oil
6 Handle financial matters, such as paying bills
7 Perform household cleaning
8 Wash, fold, and put away laundry
9 Purchase groceries
10 Earn most of the money to support the family
11 Wrap gifts (e.g. birthday or holiday presents)
12 Decorate the house
13 Shovel snow to clear driveways and sidewalks
14 Stay home with a child who is sick

Notes: Items 1, 2, 4, 5, 6, 10, and 13 were written to reflect male gender role stereotypes; items 3, 7, 8, 9,11, 12, and 14 were written to reflect female gender role stereotypes

The streamer's language style scale (Berryman-Fink & Wilcox, 1983)

Please rate the speaker on seven semantic differential items:

7-point scale:1/7

very task oriented/not at all task oriented;

very socioemotionally oriented/ not at all socioemotionally oriented;

often pronounces-ing word endings/rarely pronounces-ing word endings;

often interrupts/rarely interrupts;

very talkative/not at all talkative;

high-pitch voice/low-pitch voice;

very expressive voice/not at all expressive voice.

The streamer's professionalism scale (Ohanian, 1990)

Please rate the professionalism of the streamer

7-point scale: 1/7

Not an expert/ Expert

Inexperienced/Experienced

Unknowledgeable/Knowledgeable

Unqualified/Qualified

Unskilled/Skilled
